# Clinical characteristics of abruptly increased paediatric patients with Omicron BF.7 or BA.5.2 in Beijing

**DOI:** 10.1186/s12985-023-02177-x

**Published:** 2023-09-08

**Authors:** Lei Yu, Congying Wang, Xiaoyun Li, Xinning Wang, Yingying Kang, Xiaomei Ma, Rui Sun, Yu Sun, Runan Zhu, Liping Jia, Yao Yao, Xiaohui Li, Daitao Zhang, Yang Pan, Bing Lv, Jing Yuan, Linqing Zhao, Qinglong Gu, Jian Zhang

**Affiliations:** 1 Department of Infection Management, Children’s Hospital Capital Institute of Pediatrics, Beijing, 100020 China; 2 Department of Cardiology, Children’s Hospital Capital Institute of Pediatrics, Beijing, 100020 China; 3 Laboratory of Virology, Beijing Key Laboratory of Etiology of Viral Disease in Children, Capital Institute of Pediatrics, Beijing, 100020 China; 4 Department of Rheumatology and Immunology, Children’s Hospital Capital Institute of Pediatrics, Beijing, 100020 China; 5 Institute for Infectious Disease and Endemic Disease Control, Beijing Center for Disease Control and Prevention, Beijing, 100013 China; 6 Department of Bacteriology, Capital Institute of Pediatrics, Beijing, 100020 China; 7 Department of Otorhinolaryngology, Children’s Hospital Capital Institute of Pediatrics, No. 2 Yabao Road, Chaoyang District, Beijing, 100020 China; 8 Department of Neurosurgery, Children’s Hospital Capital Institute of Pediatrics, No. 2 Yabao Road, Chaoyang District, Beijing, 100020 China

**Keywords:** Paediatric patients, Omicron, Clinical characteristics, Risk factors

## Abstract

**Background:**

The coronavirus disease 2019 outbreak has hit Beijing since mid-Nov, 2022, with soaring growth of severe acute respiratory syndrome coronavirus 2 (SARS-CoV-2) among children. Therefore, it is vital to determine the clinical manifestations of epidemic SARS-CoV-2 strains in paediatric patients.

**Methods:**

In this study, nucleic acid tests (NATs) for SARS-CoV-2 were performed in paediatric outpatients with symptoms of acute respiratory tract infection during 18 Nov–6 Dec, 2022. Half of the outpatients positive for SARS-CoV-2 were randomly selected to screen for other respiratory pathogens, whereas those with low cycle threshold values in SARS-CoV-2 NATs were amplified and sequenced to determine the SARS-CoV-2 variants. Finally, children positive for SARS-CoV-2 with clinical information in detail were enrolled in a follow-up study to identify potential factors significantly associated with long recovery.

**Results:**

Among 9625 paediatric outpatients tested for nucleic acid of SARS-CoV-2, 733 (7.62%, 733/9625) were identified as SARS-CoV-2 NAT positive, with only three (0.82%, 3/366) co-infected with other pathogens among 366 randomly selected patients, and 71 (62.83%) determined as Omicron subvariant BF.7 and 42 (37.22%) as BA.5.2 among 113 successfully sequenced. Among the 681 patients with complete clinical information, fever was the most common symptom (96.8%). In a follow-up study of 592 patients, 46.96% became asymptomatic on the third day and 65.71% on the fifth day. Only 1.7% of infected children experienced febrile seizures. Combined with abnormal C-reactive protein, a higher percentage of antibiotics administration was observed. More co-living members and longer duration of first symptoms served as independent risk factors for long-term recovery, especially in children vaccinated for SARS-CoV-2.

**Conclusions:**

BF.7 and BA.5.2 were the dominate Omicron subvariants and caused milder infections during the SARS-CoV-2 outbreak in Beijing. The number of co-living members and duration of first symptoms were independent risk factors for long-term recovery.

**Supplementary Information:**

The online version contains supplementary material available at 10.1186/s12985-023-02177-x.

## Introduction

Severe acute respiratory syndrome coronavirus 2 (SARS-CoV-2), an RNA virus, has undergone a series of mutations and evolutions, giving rise to various variants and subvariants with strong infectious and immune escape ability, which makes coronavirus disease 2019 (COVID-19) a global pandemic that has a devastating impact on individuals and multiple aspects of our society [1–3]. On Nov, 2021, a highly mutated SARS-CoV-2 variant, Omicron (B.1.1.529), was first detected in South Africa and has rapidly spread worldwide [4].

Previous studies have pointed out that Omicron variant infection is characterised by faster transmission and stronger immune escape than those of previous variants, and by a higher risk of reinfection and milder infection among vaccinated individuals, including children [1, 5–7]. Among young patients, the comorbidities, hospitalisation rates, respiratory symptoms, severity and mortality associated with Omicron were relatively low compared to those of earlier strains [8, 9]. Globally, the World Health Organization estimates that mortality from Omicron waves in children and adolescents was < 0.4% (2506 cases) across all age groups since its outbreak [10]. Compared to other common viral respiratory tract infections in children, the respiratory symptoms after Omicron infection are more obvious [11], but the risk profile and possible sequelae of Omicron infection remain unclear [12].

Owing to the national dynamic zero-COVID strategy in China, there was no persistent local transmission of SARS-CoV-2 in Beijing before Dec, 2022. Following the adjustment of prevention and control policies for SARS-CoV-2 in mid-Nov, 2022, a round of the COVID-19 pandemic hit Beijing. A rapidly increasing number of cases have been reported in children, a special population of sporadic SARS-CoV-2 infected cases reported in Beijing previously. Therefore, it is vital to reveal the clinical manifestations of paediatric patients associated with the current epidemic SARS-CoV-2 strains.

In this study, we enrolled children confirmed as SARS-CoV-2 nucleic acid-positive with detailed clinical information to summarise the clinical manifestations of infected children and identify potential factors substantially associated with long-term recovery of clinical symptoms.

## Methods

### Ethical approval

The study protocol was reviewed and approved by the Ethics Committee of the Capital Institute of Pediatrics (Approval Number: SHERLLM2023006).

### Study design and the eligible criteria for participants

This single-centre, prospective study was conducted at Children’s Hospital of Capital Institute of Pediatrics in Beijing during 18 Nov to 6 Dec, 2022. For all paediatric outpatients with symptoms of acute respiratory tract infection (ARI), nucleic acid tests (NATs) for SARS-CoV-2 were required and half of them were screened for other respiratory pathogens. To confirm the subvariants of Omicron, specimens with low cycle threshold (Ct) values in SARS-CoV-2 NATs were subtyped, and partial subtyping results were confirmed by Meta-Genomic Next-Generation Sequencing (mNGS).

**Fig. 1:**
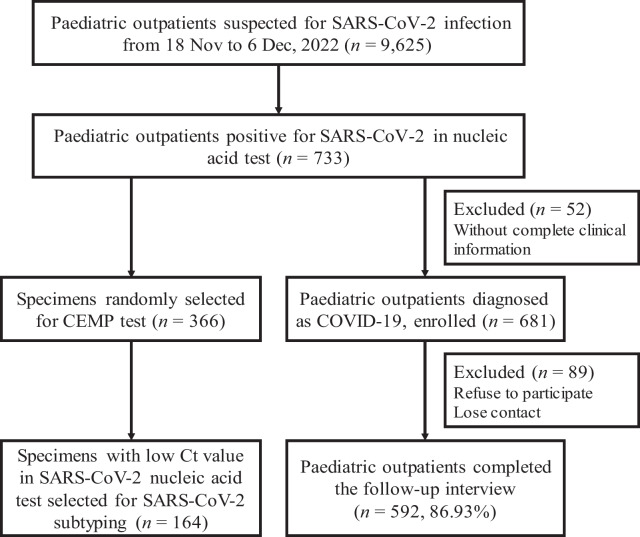
Flow chart of the study design. SARS-CoV-2, Severe acute respiratory syndrome coronavirus 2. CEMP, Capillary electrophoresis-based multiplex polymerase chain reaction assays. mNGS, Meta-genomic next-generation sequencing

The SARS-CoV-2 positive outpatients (SARS-CoV-2 group) were enrolled according to the following eligibility criteria for clinical data analyses: (i) volunteered to participate, (ii) within 3 days of first onset, (iii) Chinese descent, (iv) ≤ 18 years of age, and (v) with complete clinical data. To identify the potential factors significantly associated with the long-term recovery of clinical symptoms, trained interviewers conducted a follow-up trial (Fig. 1).

### Severe acute respiratory syndrome coronavirus 2 (SARS‑CoV‑2) nucleic acid detection

Throat swab specimens were obtained from the children upon admission and stored in inactivated virus preservation solution. Upon arrival at the laboratory, all specimens were blended for 10 s and total nucleic acids (DNA and RNA) were extracted from 200 µL of each specimen using the Nucleic Acid Extraction Kit (DAAN GENE, China). Two SARS-CoV-2 genes, ORF1ab (encoding the RNA-dependent RNA polymerase) and N (encoding the nucleocapsid protein), were amplified using SARS-CoV-2 nucleic acid detection kits (DAAN GENE, China or Shanghai BioGerm Medical Technology Co., Ltd., China), following the manufacturer’s instructions. For kits from DAAN GENE, Ct ≤ 30 was set as the cut-off value for SARS-CoV-2 positivity, and for kits from BioGerm Medical Technology, Ct ≤ 40 was the cut-off value.

### Respiratory pathogen screening

To evaluate other common respiratory infections, specimens collected from half of the paediatric patients positive for SARS-CoV-2 were randomly selected and subjected to capillary electrophoresis-based multiplex polymerase chain reaction (CEMP) (Ningbo HEALTH Gene Technologies Ltd., Ningbo, China) for 13 pathogen screening.

For CEMP test, total nucleic acid (DNA and RNA) was extracted first from 140 µL of each collected specimen using the QIAamp MinElute Virus Spin Kit (Qiagen GmbH, Germany) according to the manufacturer’s instructions. Then, nucleic acids were amplified in the reaction mixture containing PCR enzyme, 0.25 μM of each primer, dNTPs, MgCl_2_, and buffer, according to the manufacturer’s instructions for the CEMP test, and then subjected to capillary electrophoresis on a GeXP capillary electrophoresis system (Sciex, Concord, ON, Canada). Fifteen pairs of primers were used to detect 13 pathogens using human DNA and RNA as control. Signals from the 15 labelled PCR products were measured using fluorescence. As per the kit instructions, the positions of pathogen amplicons were as follows: influenza virus (Flu) A 105 nt (2009H1N1 163.3 nt, H3N2 244.9 nt), human adenovirus (HAdV) 110.2/113.9 nt (representing different subtypes), human bocavirus (HBoV) 121.6 nt, human rhinovirus (Rh) 129.6 nt, human parainfluenza virus (HPIV) 181.6 nt, *Chlamydia pneumoniae* (Cp) 190.5 nt, and human metapneumovirus (HMPV) 202.8 nt, flu B 212.7 nt, *Mycoplasma pneumoniae* (Mp) 217 nt, human coronavirus (HCoV) 265.1 nt, and respiratory syncytial virus (RSV) 280.3 nt. The remaining nucleic acids were preserved at –20 °C for future use.

### Reverse transcription and PCR for SARS-CoV-2 subtyping

The extracted nucleic acids from the CEMP assay with Ct ≤ 20 determined by the DAAN kits or ≤ Ct 30 determined by the BioGerm kits for SARS-CoV-2 nucleic acid detection were used as templates to synthesise cDNA using a conventional two-step reverse transcription reaction with random primers according to the manufacturer’s instructions. Moloney Murine Leukaemia virus (M-MLV) reverse transcriptase (200 U/μL) (Invitrogen, USA), Ribonuclease Inhibitor (50 U/μl, TransGen Biotech, China), and dNTP (10 mM, TransGen Biotech, China) were added separately to reaction mixtures.

To obtain the spike gene sequences for SARS-CoV-2 subtyping of Omicron, 5 μL cDNA obtained by reverse transcription was used as a template in a round of PCR using forward (S1:5’-AAT CTT AGG GAA TTT GTG TT-3’) and reverse (S2:5’-AGT ACT ACT ACT CTG TAT GG-3’) primers to get 976 bp products. All PCRs were performed in a 25 µL final volume mixture according to the kits 2 × EasyTaq® PCR SuperMix and manufacturer’s instructions (TransGen Biotech, China). Amplified products were visualised by electrophoresis on 2% agarose gels stained with ethidium bromide. The amplified products were sequenced by SinoGenoMax (Beijing, China).

### Phylogenetic analyses

Lasergene’s DNA SeqMan software (version 7.1.0; DNA Star Inc. Madison, WI, USA) was used to assemble nucleotide sequences. MAFFT software was used to align the sequences. Phylogenetic trees were constructed using the maximum likelihood method and the Kimura 2-parameter model with 1000 bootstrap pseudo-replicates in MEGA VI. The reference sequences (Additional file 1: Table S1) were obtained from the National Centre for Biotechnology Information.

### Meta-genomic next-generation sequencing (mNGS)

To confirm the results of the phylogenetic analyses, 13 specimens were sent to the Beijing Center for Disease Control and Prevention for mNGS as previously described [13]. In brief, viral RNA was extracted from 200 µL of sample and eluted in 90 µL elution buffer by the KingFisher Flex Purification System (Thermo Fisher, USA). cDNA was synthesised from the extracted RNA using random hexamers and LunaScript RT SuperMix Kit (New England Biolabs, UK) according to the manufacturer’s instructions. Subsequently, the revised ARTIC network SARS-CoV-2 V4.1 primer scheme and Q5 High-Fidelity DNA polymerase (New England Biolabs, UK) were used for SARS-CoV-2 whole-genome multiplex PCR amplification. The PCR products were used to prepare a library for mNGS using a Nextera XT DNA Sample Preparation and Index kit and DNA Prep Sample Preparation and Index kit (Illumina, San Diego, CA, USA) following the manufacturer’s instructions and sequenced on an Illumina MiSeq or MiniSeq platform using the 2 × 150 cycles paired-end sequencing protocol.

### Clinical data collection

At baseline, demographic characteristics and clinical manifestations of the study children were collected using a Case report form (CRF), including age, sex, date of symptom onset, major symptoms, routine blood tests, and vaccination status. Routine blood test items were extracted from the patient record systems, including white blood cell count, lymphocyte ratio, monocyte ratio, neutrophil ratio, lymphocytes, monocytes, leukocytes, red blood cells, haemoglobin, platelets, eosinophils, basophilic granulocytes, and C-reactive protein (CRP). Children vaccinated with one to three doses of SARS-CoV-2 inactivated vaccines, including Sinopharm BBIBP-CorV (Beijing Institute of Biological Products Co., Ltd.), Sinopharm WIBP-CorV (Wuhan Institute of Biological Products Co., Ltd.) and CoronaVac (Sinovac Life Sciences Co., Ltd.), were defined as “children with SARS-CoV-2 vaccination”, while children had not been vaccinated with SARS-CoV-2 vaccines were defined as “children without SARS-CoV-2 vaccination”.

In one week following the visiting of hospital, supervisors of children were contacted via telephones to inquire for the type, number and duration of symptoms, drug regimens (including antibiotics, traditional Chinese medicine, and antifebriles), none-drug treatment (including drinking lots of water, eating fresh fruits, and taking additional vitamin C) of children, as well as SARS-CoV-2 infection and symptoms of family members. The follow-up trial was completed by trained interviewers, and the information was typed into a predesigned CRF. In addition, perinatal situations were recorded.

### Statistical analysis

Demographic and clinical data were expressed as median (interquartile range, IQR) or mean ± standard deviation (SD) for continuous variables and as count (percentage, %) for categorical variables. Potential factors associated with long (long recovery group, ≥ 3 days) or short (short recovery group, < 3 days) recovery time of clinical symptoms and SARS-CoV-2 infection risks were identified using logistic regression analyses before and after adjusting for confounders. The Shapiro–Wilk test was used to test the normality of continuous variables. Comparisons between different groups were performed using the Wilcoxon rank sum test for continuous variables with skewed distributions, and the χ^2^ test or Fisher’s exact test for categorical variables. Effect sizes were expressed as odds ratios (OR) and 95% confidence intervals (CI). All probabilities were 2-sided, and *P* values less than 0.05 were considered statistically significant. All statistical analyses were performed under the R programming environment (version 4.0.3).

## Results

### SARS-CoV-2 and co-pathogen infection

**Fig. 2:**
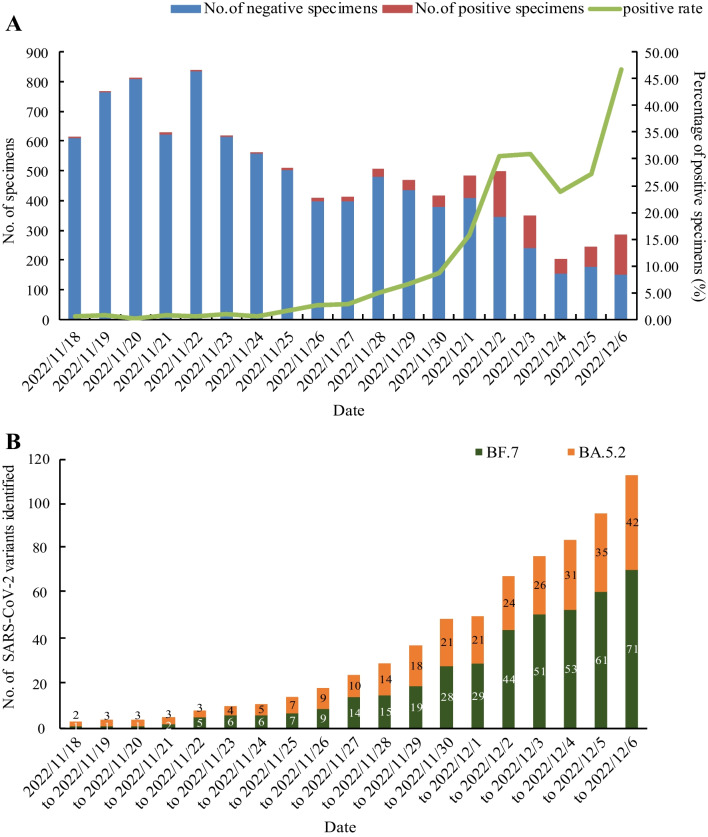
Distribution of SARS-CoV-2 positive specimens from paediatric outpatients during 18 Nov-6 Dec, 2022. **A** Number or percentage of SARS-CoV-2 positive specimens per day. **B** The cumulative positive cases identified as Omicron subvariant BA.5.2 or BF. 7 per day

During 18 Nov–6 Dec, 2022, 9625 paediatric outpatients with symptoms of ARI were tested for SARS-CoV-2 NATs, and 733 were confirmed to be SARS-CoV-2 positive (7.62%, 733/9625) (Fig. 1). The first SARS-CoV-2 positive paediatric case was detected in 18 Nov, 2022 (0.48%, 3/615), and the positivity rate of SARS-CoV-2 remained low (from 0.12 to 0.97%) in the following days. While the number of positive paediatric cases increased obviously from Nov 25 to 30 (from 1.57 to 8.65%), an abrupt increase (15.73%, 76/483) was observed from Dec 1, to its peak on 6 Dec, 2022 (46.67%, 133/285) (Fig. 2A).

Among the 733 SARS-CoV-2 positive specimens, 366 were randomly chosen for respiratory pathogen screening using the CEMP test. Only three patients (0.82%) were coinfected with other pathogens (one with HAdV, one with HBoV, and one with Mp).

### BF.7 and BA.5.2 dominated in SARS-CoV-2 subtyping

Among the 366 specimens, 164 had low Ct values for SARS-CoV-2 NATs, of which 113 sequences of spike gene were harvested through amplification and sequencing. By further phylogenetic analyses, 71 cases were determined as BF.7 sub-variants (62.83%, 71/113) and 42 as BA.5.2 sub-variants (37.17%, 42/113) of Omicron (Fig. 2B). The subtyping results of 13 specimens selected were confirmed by mNGS with the same results as the phylogenetic analyses (Additional file 1: Fig. S1).

### Clinical manifestations of children with SARS-CoV-2 infection

**Fig. 3:**
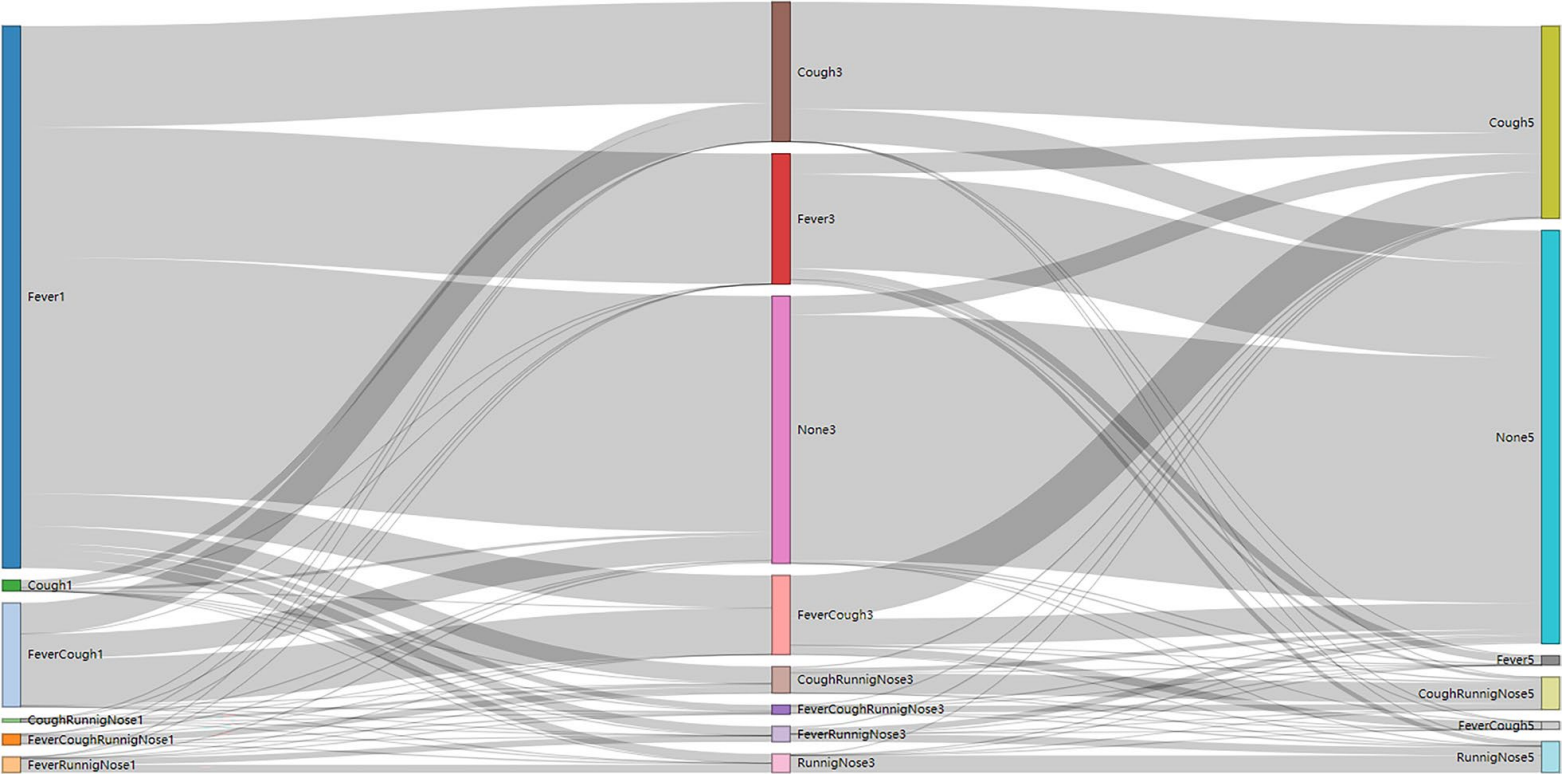
Changes in the major symptoms at the first, third, and fifth days from the onset of SARS-CoV-2 infection. xx1, xx3, or xx5, xx symptoms on the first, third, or fifth day

Among 733 paediatric outpatients confirmed as SARS-CoV-2 infection, 681 cases with complete clinical information were enrolled in the study (Fig. 1), with the median age of 3.4 (IQR [1.6–8.5]) years. As for the first onset symptom of 681 outpatients, fever (≥ 37.5℃) accounted for 96.8%, while cough accounted for 23.2%, sore throat 6.4%, rhinorrhoea 5.5%, vomiting 4.9%, and febrile seizures 1.7%. The distribution of clinical manifestations within 5 days of symptom onset revealed that 95.5% of children started with fever on the first day, of which 95% had fever that resolved within the first 3 days, and 30% of children had a combined cough over the course of the first 5 days (Table 1). The major symptom on the first day was fever, which turned into a cough on the third day. On the third day, 46.96% of patients showed no symptoms, which increased to 65.71% on the fifth day (Fig. 3).

**Table 1:** Clinical symptoms within the first 5 days from the onset of Omicron infection among paediatric outpatients

Symptoms	Day 1	Day 2	Day 3	Day 4	Day 5
Fever	95.5% (567/594)	78.6% (440/560)	31.1% (174/559)	5.0% (28/555)	2.3% (13/555)
Cough	16.2% (96/594)	25.5% (143/560)	30.9% (173/559)	31.7% (176/555)	28.5% (158/555)
Sore throat	5.9% (35/594)	5.5% (31/560)	4.8% (27/559)	3.4% (19/555)	2.0% (11/555)
Vomiting	4.0% (24/594)	2.5% (14/560)	0.9% (5/559)	0% (0/555)	0.2% (1/555)
Rhinorrhoea	3.4% (20/594)	5.0% (28/560)	8.4% (47/559)	8.1% (45/555)	7.7% (43/555)
Diarrhoea	2.0% (12/594)	1.8% (10/560)	0.9% (5/559)	0.4% (2/555)	0.4% (2/555)
Febrile seizure	1.7% (10/594)	0% (0/560)	0% (0/559)	0% (0/555)	0% (0/555)
Headache	1.5% (9/594)	1.6% (9/560)	0.4% (2/559)	0% (0/555)	0% (0/555)
Nasal obstruction	1.3% (8/594)	1.8% (10/560)	2.0% (11/559)	2.2% (12/555)	1.8% (10/555)
Myalgia	1.2% (7/594)	0.9% (5/560)	0.2% (1/559)	0% (0/555)	0% (0/555)
Dizziness	0.5% (3/594)	0.4% (2/560)	0.2% (1/559)	0.2% (1/555)	0.2% (1/555)
Milk regurgitation	0.2% (1/594)	0.2% (1/560)	0% (0/559)	0% (0/555)	0% (0/555)
Sneeze	0% (0/594)	0.2% (1/560)	0.4% (2/559)	0.4% (2/555)	0.4% (2/555)
Stomach-ache	0.7% (4/594)	0.5% (3/560)	0.4% (2/559)	0.2% (1/555)	0.2% (1/555)
Drowsiness	0.5% (3/594)	0.5% (3/560)	0.2% (1/559)	0.2% (1/555)	0% (0/555)
Laryngitis	0.2% (1/594)	0.2% (1/560)	0.2% (1/559)	0% (0/555)	0% (0/555)
Weakness	0.7% (4/594)	0.9% (5/560)	0.7% (4/559)	0.5% (3/555)	0.5% (3/555)
Loss of taste	0% (0/594)	0.2% (1/560)	0.4% (2/559)	0.4% (2/555)	0.4% (2/555)
Loss of smell	0% (0/594)	0.2% (1/560)	0.4% (2/559)	0.4% (2/555)	0.4% (2/555)
Sputum in throat	0.2% (1/594)	0.2% (1/560)	0.2% (1/559)	0% (0/555)	0% (0/555)
Jaundice	0.2% (1/594)	0.2% (1/560)	0.2% (1/559)	0.2% (1/555)	0.2% (1/555)
Trachyphonia	0.8% (5/594)	0.7% (4/560)	0.4% (2/559)	0% (0/555)	0% (0/555)
Whoop	0.2% (1/594)	0.2% (1/560)	0% (0/559)	0% (0/555)	0% (0/555)
Nausea	0.5% (3/594)	0.5% (3/560)	0% (0/559)	0% (0/555)	0% (0/555)
Chest distress	0.3% (2/594)	0.2% (1/560)	0% (0/559)	0% (0/555)	0% (0/555)

Data are expressed as percentage

Considering the impact of CRP value, there were 180 SARS-CoV-2 infected children (26.43%, 180/681) with abnormal CRP (≥ 8 mg/dL), while more (73.57%, 501/681) were with normal CRP (< 8 mg/dL). The percentage of non-use of antibiotics in children with normal CRP levels (87.8%) was significantly higher than that in children with abnormal CRP levels (73.3%) (*P* < 0.001). However, no significant difference was observed in clinical symptoms at the first visit or at the time of symptom remission (Additional file 1: Table S2).

### Risk factors of long-term recovery of clinical symptoms

Among 681 cases enrolled, 592 (362 boys and 230 girls) outpatients, with the median age of 3.5 (IQR [1.6–8.7]) years, completed the follow-up interview with a response rate of 86.93% (Fig. 1). Upon stratification by recovery days, 592 children were divided into two groups: a short (< 3 days) group (n = 216, 36.49%) and a long-term recovery (≥ 3 days) group (n = 376, 63.51%).

Significant differences between short and long-term recovery groups included these indicators: co-livings infected with SARS-CoV-2, white blood cell, leukocyte, antibiotics administration, intake of traditional Chinese medicine (TCM) drugs, intake of febrifuge, and eating fresh fruits (*P* < 0.05) (Table 2).

**Table 2:** Demographic and clinical characteristics of paediatric outpatients infected with SARS-CoV-2 and stratified by recovery days

Baseline characteristics	Short recovery group (n = 216)	Long recovery group (n = 376)	*P*
Age (years)	3.1 [1.5, 7.8]	3.7 [1.7, 9.2]	0.135
Boys	135 (62.5)	227 (60.4)	0.672
Number of co-living members	3 [2, 4]	3 [2, 4]	0.959
Co-livings infected with SARS-CoV-2	158 (77.5)	315 (85.8)	0.015
Premature at birth	5 (4.1)	5 (3.0)	0.861
Respiratory distress at birth	1 (0.8)	2 (1.3)	0.999
Regular immunization	212 (99.1)	370 (98.7)	0.999
Previous severe diseases	8 (3.7)	20 (5.3)	0.505
White blood cell (10^9/L)	6.26 [4.53, 7.90]	6.61 [5.03, 8.56]	0.018
Lymphocyte ratio	0.20 [0.11, 0.31]	0.19 [0.12, 0.28]	0.467
Monocyte ratio	0.11 [0.08, 0.14]	0.10 [0.08, 0.13]	0.102
Neutrophil ratio	0.69 [0.52, 0.78]	0.70 [0.58, 0.78]	0.239
Lymphocyte (10^9/L)	1.11 [0.76, 1.64]	1.20 [0.76, 1.76]	0.3
Monocyte (10^9/L)	0.64 [0.49, 0.82]	0.68 [0.52, 0.85]	0.21
Leukocyte (10^9/L)	3.88 [2.49, 5.65]	4.43 [2.96, 6.20]	0.008
Red blood cell (10^12/L)	4.66 [4.34, 5.00]	4.72 [4.45, 5.01]	0.134
Haemoglobin (g/L)	129.5 [121, 139]	130 [120.75, 140]	0.734
Platelet (10^9/L)	214 [187, 253]	213 [184, 253.25]	0.953
Eosinophils (10^9/L)	0.02 [0.01, 0.08]	0.02 [0.01, 0.06]	0.878
Basophilic granulocyte (10^9/L)	0.01 [0.01, 0.02]	0.01 [0.01, 0.02]	0.933
C-reactive protein (mg/L)	4 [1.1, 8]	4 [1.48, 8]	0.793
Antibiotics administration	27 (12.6)	87 (23.2)	0.002
Intake of TCM drugs	121 (59.9)	271 (76.6)	< 0.001
Intake of febrifuge	177 (85.5)	339 (94.4)	0.001
Drinking lots of water	78 (37.1)	119 (31.9)	0.233
Eating fresh fruits	21 (10.0)	8 (2.1)	< 0.001

Data were expressed as median [interquartile range] or count (percentage, %) and were compared using the Wilcoxon rank-sum test, χ^2^ test or Fisher’s exact test

*SARS-CoV-2* Severe acute respiratory syndrome coronavirus 2, *TCM* Traditional Chinese medicine

Significant risk factors for long-term recovery from clinical symptoms were further analyzed using univariate logistic regression analysis (Table 3). Among children without SARS-CoV-2 vaccination, the number of co-living members and remission time of the first symptom were significant risk factors for prolonged recovery (OR [1.09] and [1.24];* P* = 0.011 and < 0.001, respectively), whereas only the remission time of the first symptom remained statistically significant (OR [1.56]; 95% CI [1.30–1.88]; *P* < 0.001) after adjusting for confounders. In contrast, co-living members, birth weight, and remission time of the first symptom among children vaccinated for SARS-CoV-2 were all identified as significant risk factors for long-term recovery of clinical symptoms, even after adjusting for confounders. For instance, per 1 d increment in remission time of first symptom was associated with 1.80-fold increased risk of long-term recovery after adjustment (95% CI [1.44–2.24]; *P* < 0.001).

**Table 3:** Risk factors for long-term recovery of clinical symptoms

Risk factors	Model 1			Model 2		
	OR	95% CI	*P*	OR	95% CI	*P*
*Children without SARS-CoV-2 vaccination*						
Co-living members	1.09	1.02–1.16	0.011	1.07	0.96–1.18	0.223
Remission time of first symptom	1.24	1.12–1.37	< 0.001	1.56	1.30–1.88	< 0.001
*Children with SARS-CoV-2 vaccination*						
Co-living members	1.27	1.17–1.38	< 0.001	1.28	1.12–1.45	< 0.001
Birth weight	1.24	1.05–1.46	0.010	1.38	1.05–1.81	0.020
Remission time of first symptom	1.55	1.35–1.78	< 0.001	1.80	1.44–2.24	< 0.001

*OR* Odds ratio, *95% CI* 95% Confidence interval, *Model 1* Without adjustment, *Model 2* Adjusted for confounders

## Discussion

Following the first SARS-CoV-2 positive paediatric patient identified on Nov 18, 2022, an outbreak of SARS-CoV-2 in children was confirmed in Beijing. Surprisingly, in routine paediatric practice, our team identified 733 SARS-CoV-2 outpatients within only 19 days from a single children’s hospital in Beijing, while rare co-infections with other respiratory pathogens were detected. Among the 113 sequences harvested in this study, the Omicron subvariants BF.7 and BA.5.2 were the predominant ones. Therefore, children also were evolved while BA.5.2 and BF.7 were dominant in Beijing during this outbreak of Omicron, accounting for 90% of local cases since Nov 14 [13].

Omicron variants have been classified as variants of concern (VOC) because their infectivity is over tenfold higher than that of the original virus [14]. From the perspective of virology, Omicron contains 32 mutations in the spike protein located mostly in the N-terminal and receptor-binding domains, which can enhance its ability to bind antibodies [15]. The BA.5.2 variant was composed of two branches, BA.5.2.48 and BA.5.2.49, by PANGO with high-frequency mutations unique to the BA.5.2 variant circulating in China (ORF1b: T1050N in BA.5.2.48 and Spike: T883I in BA.5.2.49), while the BF.7 variant prevalent in China, named BF.7.14 by PANGO, was an independent evolutionary branch of Omicron with three unique non-synonymous mutations (NS7a:H47Y, NSP2:V94L, and Spike:C1243F) [16]. The Omicron subvariants BF.7 and BA.5.2 have been reported to enhance their transmissibility [17], and BF.7 might be more infectious due to its greater rate of antigen-receptor binding than BA.5.2 [18]. However, few clinical manifestations have been observed in children infected with Omicron subvariant BF.7 or BA.5.2 in Beijing, China.

In this pilot survey of the Omicron outbreak in Beijing, we summarised the clinical manifestations of children infected with Omicron subvariant BF.7 or BA.5.2, as well as the factors associated with long-term recovery of clinical symptoms and Omicron infection. It is worth noting that fever was the most common symptom among children infected with the Omicron subvariant BF.7 or BA.5.2, followed by cough. After comparing the symptom spectrum over 5 days of first onset, we found that 46.96% became non-symptomatic on the third day, and 65.71% became non-symptomatic on the fifth day. These results support the idea that the variant Omicron of SARS-CoV-2 causes milder infection [19], even during its evolution from BA.1 to BF.7.

Neurological symptoms are common in adults with COVID-19 [20]. In contrast, in this study, only 1.7% of the infected children experienced febrile seizures, which may, at least in part, be explained by differences in the pathogenicity of Omicron variants in children.

Evidence from adults has revealed that antibiotic prescriptions for combatting COVID-19 seemed unnecessary [21]. In this study, we explored the administration of antibiotics in children infected with Omicron subvariants BF.7 or BA.5.2 in the early stages, based on CRP, a marker of the inflammatory response. Combined with the abnormal CRP, a higher percentage of antibiotics was observed. Among those outpatients with long-term recovery, more antibiotics, TCM drugs and anti-febrifuge were used. Therefore, the CRP value indicated the use of antibiotics in clinical practice, regardless of whether significant differences were observed between the short and long recovery groups. Given the comparable number of symptoms at the first visit and the similar intervals for symptom remission between infected children with normal and abnormal CRP levels, it is suggested that the increase in CRP in the early stages of Omicron subvariant BF.7 or BA.5.2 infection may result from a systemic inflammatory response. Therefore, antibiotics used in paediatric patients infected with the Omicron subvariant BF.7 or BA.5.2 should also be unnecessary.

To identify potential risk factors for long-term recovery after Omicron subvariant BF.7 or BA.5.2 infection in children, the results of the study indicated that more co-living members and a longer duration of first symptoms served as independent risk factors for prolonged disease intervals. Especially in children with SARS-CoV-2 vaccination, the number of co-living members served as an independent risk factor for long-term recovery, even after adjustment, which can be explained by higher likelihood of mutual Omicron infectivity with more co-living members [22–24]. The results also can explain our previous results that the infection rate of SARS-CoV-2 was low among children in Beijing with no family clustering or close contact [25]. These results strengthen the urgent need for self-isolation to prevent family transmission for the primary prevention of Omicron infection in children, as well as the avoidance of mass-gathering events and contact with high-risk persons. Among the first symptoms with a longer duration, another indicator of long-term recovery, fever occurred in the vast majority of cases infected by Omicron subvariant BF.7 or BA.5.2 in clinical practice.

## Conclusions

The results of this study revealed that the Omicron subvariant BF.7 and BA.5.2 were the dominant viral pathogens in this epidemic wave, while fever was the most common symptom among the infected children, and more co-living members and longer duration of first symptoms were independently associated with a higher risk of prolonged disease intervals. A note of caution should be taken in the interpretation of the results of this study, considering the limited number of accessible children from a single centre in Beijing. Further studies of the clinical characteristics of different Omicron variant infections are required. However, this is the first report on the clinical manifestations in children infected with the Omicron subvariants BF.7 or BA.5.2 in China. Our pilot survey provides an anchoring point for a better understanding of the clinical manifestations and risk profiles of Omicron subvariant BF.7 or BA.5.2 infection in children and has far-reaching implications in routine clinical practice.

### Supplementary Information

**Additional file 1: Table S1.** Reference sequences downloaded from the National Center for Biotechnology Information. **Table S2.** Comparison of drug regimens and clinical symptoms among SARS-CoV-2 infected children stratified by CRP. **Fig. S1.** Phylogeny tree of S gene segments from SARS-CoV-2 positive specimens built by Maximum likelihood method.

## Data Availability

The datasets used and/or analyzed in this study are obtained and available from the corresponding authors upon a reasonable request.

## Abbreviations

COVID-19: Coronavirus disease 2019
SARS-CoV-2: Severe acute respiratory syndrome coronavirus 2
NATs: Nucleic acid tests
ARI: Acute respiratory tract infection
WHO: World Health Organization
mNGS: Meta-genomic next-generation sequencing
Ct: Cycle threshold
PCR: Polymerase chain reaction
CEMP: Capillary electrophoresis-based multiplex polymerase chain reaction
Flu: Influenza virus
HAdV: Human adenovirus
HBoV: Human bocavirus
Rh: Human rhinovirus
HPIV: Human parainfluenza virus
Cp: *chlamydia* *pneumoniae*
HMPV: Human metapneumovirus
Mp: *Mycoplasma pneumoniae*
HCoV: Human coronavirus
RSV: Respiratory syncytial virus
CRF: Case report form
CRP: C-reactive protein
IQR: Interquartile range
SD: Standard deviation
OR: Odds ratio
CI: Confidence interval
TCM: Traditional Chinese medicine
VOC: Variant of concern
