# Supplementary material for: Clinical characteristics of abruptly increased paediatric patients with Omicron BF.7 or BA.5.2 in Beijing
Source: Virol J. 2023 Sep 8;20:209. doi: 10.1186/s12985-023-02177-x (PMC10492331; doi:10.1186/s12985-023-02177-x)

**Supplementary Table 1.** Reference sequences downloaded from the National Center for Biotechnology Information.

| **NCBI Number** | **Isolation definition** | **Variants** | **Genome length (bp)** | **Loci of S gene** |
| --- | --- | --- | --- | --- |
| NC_045512.2 | Wuhan-Hu-1 | B | 29903 | 21563..25384 |
| OK538014.1 | SARS-CoV-2/human/BTN/RCDC0011-SV210149/2021 | alpha B.1.1.7 | 29804 | 21516..25328 |
| OP522609.1 | SARS-CoV-2/human/ZAF/NHLS-UCT-GS-BM52/2021 | beta B.1.351 | 29758 | 21504..25316 |
| OP765035.1 | SARS-CoV-2/human/USA/TESTNE_A2CTYJD/2021 | gamma P.1 | 29705 | 21500..25321 |
| OP782307.1 | SARS-CoV-2/human/IND/VRDL THENI/2021 | delta B.1.617.2 | 29802 | 21538..25359 |
| OP825143.1 | SARS-CoV-2/human/TWN/KMUH-17/2022 | omicron BA.1 | 29889 | 21552..25382 |
| OP825152.1 | SARS-CoV-2/human/TWN/KMUH-26/2022 | omicron BA.2 | 29888 | 21551..25381 |
| OQ109633.1 | SARS-CoV-2/human/USA/IN-CDC-LC0959693/2022 | omicron XBB.1 | 29723 | 21515..25324 |
| OP808388.1 | SARS-CoV-2/human/USA/WA-CDC-UW22102874690/2022 | omicron BF.7 | 29745 | 21455...25261 |
| OQ204626.1 | SARS-CoV-2/human/USA/SC-CDC-UW22122629989/2022 | omicron BF.7 | 29666 | 21455...25258 |
| OQ204622.1 | SARS-CoV-2/human/USA/WA-CDC-UW22122632430/2022 | omicron BF.7 | 29666 | 21455...25258 |
| OQ204653.1 | SARS-CoV-2/human/USA/WA-CDC-UW22122652119/2022 | omicron BA.5.2 | 29669 | 21455...25261 |
| OQ244562.1 | SARS-CoV-2/human/USA/AZ-CDC-STM-UNKREPVS8/2022 | omicron BA.5.2 | 29822 | 21554..25360 |
| OQ204705.1 | SARS-CoV-2/human/USA/WA-CDC-UW22122742910/2022 | omicron BA.5.2 | 29745 | 21455...25261 |

**Supplementary Table 2.** Comparison of drug regimens and clinical symptoms among SARS-CoV-2 infected children stratified by CRP.

| **Therapies and symptoms** | **CRP < 8 mg/dL**  **(n=501)** | **CRP ≥ 8 mg/dL**  **(n=180)** | ***P*** |
| --- | --- | --- | --- |
| Clinical symptoms at the first visit |  |  | 0.676 |
| Fever | 276 (66.7) | 89 (61.4) |  |
| Fever, cough | 113 (27.3) | 47 (32.4) |  |
| Fever, cough, sore throat | 23 (5.6) | 8 (5.5) |  |
| Fever, cough, sore throat, vomiting | 2 (0.5) | 1 (0.7) |  |
| Non-use of antibiotics | 438 (87.8) | 132 (73.3) | <0.001 |
| Time of symptom remission (days) | 5.56 (0.29) | 5.05 (0.52) | 0.367 |

SARS-CoV-2, Severe acute respiratory syndrome coronavirus 2. CRP, C-reactive protein.

Data were expressed as mean (standard deviation) or count (percentage, %), and are compared using the Wilcoxon rank sum test or χ^2^ test or Fisher’s exact test.

**Supplementary Fig 1.** Phylogeny tree of S gene segments from SARS-CoV-2 positive specimens built by Maximum likelihood method.


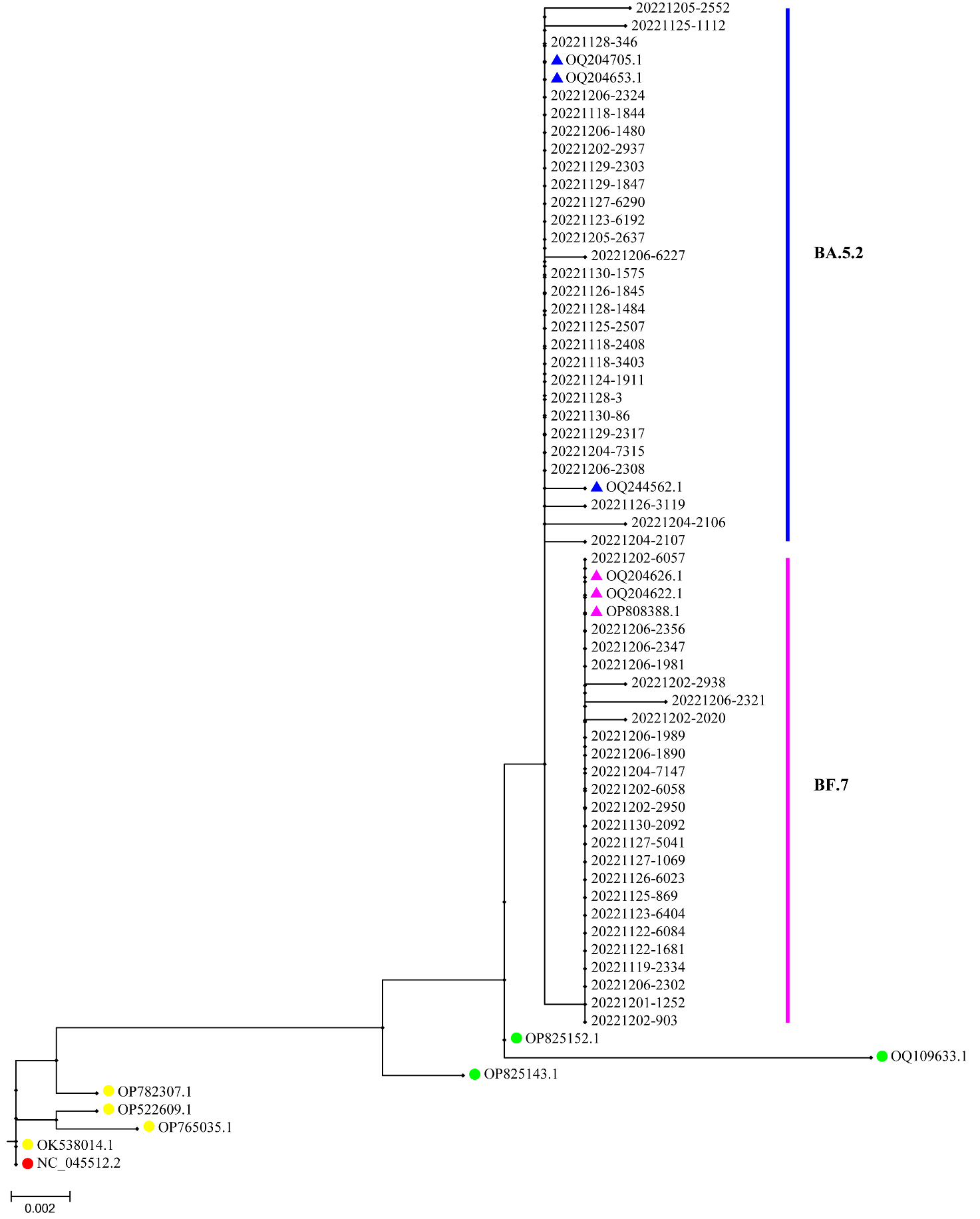

Supplement: Supplementary file 1 — Additional file 1: Table S1. Reference sequences downloaded from the National Center for Biotechnology Information. Table S2. Comparison of drug regimens and clinical symptoms among SARS-CoV-2 infected children stratified by CRP. Fig. S1. Phylogeny tree of S gene segments from SARS-CoV-2 positive specimens built by Maximum likelihood method. [file 12985_2023_2177_MOESM1_ESM.docx]
